# Tryptase Activation of Immortalized Human Urothelial Cell Mitogen-Activated Protein Kinase

**DOI:** 10.1371/journal.pone.0069948

**Published:** 2013-07-29

**Authors:** John O. Marentette, Paul J. Hauser, Robert E. Hurst, David J. Klumpp, Alice Rickard, Jane McHowat

**Affiliations:** 1 Department of Pathology, Saint Louis University School of Medicine, Saint Louis, Missouri, United States of America; 2 Department of Urology, Oklahoma University Health Sciences Center, Oklahoma City, Oklahoma, United States of America; 3 Department of Urology, Northwestern University, Feinberg School of Medicine, Chicago, Illinois, United States of America; Northwestern University, United States of America

## Abstract

The pathogenesis of interstitial cystitis/painful bladder syndrome (IC/PBS) is multifactorial, but likely involves urothelial cell dysfunction and mast cell accumulation in the bladder wall. Activated mast cells in the bladder wall release several inflammatory mediators, including histamine and tryptase. We determined whether mitogen-activated protein (MAP) kinases are activated in response to tryptase stimulation of urothelial cells derived from human normal and IC/PBS bladders. Tryptase stimulation of normal urothelial cells resulted in a 2.5-fold increase in extracellular signal regulated kinase 1/2 (ERK 1/2). A 5.5-fold increase in ERK 1/2 activity was observed in urothelial cells isolated from IC/PBS bladders. No significant change in p38 MAP kinase was observed in tryptase-stimulated normal urothelial cells but a 2.5-fold increase was observed in cells isolated from IC/PBS bladders. Inhibition of ERK 1/2 with PD98059 or inhibition of p38 MAP kinase with SB203580 did not block tryptase-stimulated iPLA_2_ activation. Incubation with the membrane phospholipid-derived PLA_2_ hydrolysis product lysoplasmenylcholine increased ERK 1/2 activity, suggesting the iPLA_2_ activation is upstream of ERK 1/2. Real time measurements of impedance to evaluate wound healing of cell cultures indicated increased healing rates in normal and IC/PBS urothelial cells in the presence of tryptase, with inhibition of ERK 1/2 significantly decreasing the wound healing rate of IC/PBS urothelium. We conclude that activation of ERK 1/2 in response to tryptase stimulation may facilitate wound healing or cell motility in areas of inflammation in the bladder associated with IC/PBS.

## Introduction

Interstitial cystitis/painful bladder syndrome (IC/PBS) is a debilitating disease associated with recurrent discomfort or pain in the bladder and the surrounding pelvic region. The pathogenesis of IC/PBS is likely multifactorial, with current proposed etiologies including urothelial cell dysfunction [1], immunologic abnormalities [2], mast cell involvement [3], neurogenic causes [4] and inhibition of urothelial cell growth by antiproliferative factor (APF) [5]. Urothelial cell dysfunction in IC/PBS is thought to initiate or mediate the events that lead to pain and bladder dysfunction observed in the disease [2], [3]. For example, increased urothelial permeability leads to diffusion of urine contents such as potassium into the bladder wall, which can depolarize nerve and muscle and cause direct tissue injury [6].

Bladder mast cell accumulation and activation plays a central role in a subset of patients with IC/PBS [3], [7]. Mast cells are more consistently increased in classic IC/PBS with Hunner’s ulcers [7], [8]. In nonulcer IC/PBS, reports on mast cell numbers show large standard deviations, possibly due to heterogeneous patient subgroups. Mast cell accumulation in IC/PBS has been associated with bladder pain [9], apoptosis [10] and detrusor fibrosis [11]. Increased urinary concentrations of histamine and tryptase are common indicators of mast cell degranulation. Mast cells may be activated by a number of mechanisms within the bladder wall that may be a direct result of increased urothelial permeability or release of neuropeptides and neurotransmitters [12], [13]. Instillation of substance P causes neurogenic inflammation and induces cystitis which is abrogated in mast cell deficient mice, suggesting that mast cells modulate bladder inflammation [14], [15]. Activation of mast cells within the bladder wall results in the release of several preformed inflammatory mediators, including histamine, cytokines, proteases such as chymase and tryptase, heparin and phospholipases. Tryptase cleaves and activates the protease-activated receptor (PAR)-2 on the endothelial cell surface [16], [17]. We have determined that tryptase stimulation of immortalized urothelial cells isolated from normal and IC/PBS bladders resulted in activation of calcium-independent phospholipase A_2_ (iPLA_2_) [18]. In previous studies, mitogen-activated protein kinases (MAP kinases) have been implicated in PLA_2_ phosphorylation and activation [19], [20]. Conversely, activation of PLA_2_ and the resultant production of membrane phospholipid-derived metabolites have been demonstrated to activate downstream MAP kinases [21], [22]. In this study, we proposed to investigate whether iPLA_2_ activation was mediated via MAP kinases in tryptase stimulated immortalized urothelial cells.

## Methods

### Culture of Bladder Urothelial Cells

Human urothelial cells (HUC) were obtained from ScienCell Research Laboratories (Carlsbad, CA), cell isolations from 3 separate donors were used. Urothelial cells isolated from normal bladder (4 separate donors) and the bladder of patients with IC/PBS (4 separate donors) were immortalized with HPV type 16E6E7 as described previously [23]. Samples were obtained from IC/PBS patients by biopsy or bladder washing during cystoscopy. Samples were collected according to an IRB-approved protocol at the Oklahoma University Health Sciences Center or at Northwestern University following informed written consent from the patient or next of kin. Cells were fixed and characterized for an anti-epithelial cytokeratin AE1/AE3 mixture based upon our previously described method [24]. Samples were viewed and images captured by confocal microscopy (MRC 1024; BioRad, Hercules, CA).

Expanded cultures were grown in EpiLife Media (Cascade Biologics, Inc. Portland, OR) with calcium (0.06 mM), growth factor supplements provided by the manufacturer and penicillin (20 U/ml)/streptomycin (100 mg/ml) (Sigma Chemical Company, St.Louis, MO). After reaching confluence, cells were grown in the same medium with 10% fetal bovine serum (FBS) and additional 1.0 mM calcium. All experiments were conducted 3 days after calcium and FBS addition. In a previous study, we have demonstrated that immortalized cells differentiate into a stratified epithelial culture with thin, tightly opposed apical superficial cells and more loosely connected underlying cells after 3 days of additional calcium and FBS incubation. These cells in culture show expression of adherens junctions, tight junctions and claudins [24].

### Urothelial Cell Stimulation

Lysoplasmenylcholine (lysoPlsCho, 5 µM) or rhSkin β-tryptase (20 ng/mL) (Promega, Madison, WI) were diluted with medium, added to the urothelial cells and the plate gently rotated to ensure thorough mixing and even distribution of stimulant across the urothelial cell layer. LysoPlsCho was prepared by alkaline hydrolysis of bovine heart choline glycerophospholipids as described previously [25]. Where appropriate, bromoenol lactone (BEL, 5 µM), PD98059 (2 µM) and SB203580 (2 µM) (all from Cayman Chemical Company, Ann Arbor, MI) were diluted in medium and added prior to tryptase stimulation.

### Measurement of MAP Kinase Activity

Extracellular signal-regulated kinase (ERK 1/2) and p38 MAP kinase activities in urothelial cells were assayed using assay kits from New England Biolabs Inc. (Beverly, MA). Assay of active p44/42 MAP kinase involves immunoprecipitation of cytosolic activated kinase with an immobilized phospho p44/42 MAP Kinase monoclonal antibody, measurement of activity by phosphorylation of Elk 1 and detection of phosphorylated Elk 1 by immunoblotting using an anti phospho Elk 1 antibody and densitometric analysis. Assay of active p38 MAP kinase involves immunoprecipitation with immobilized phospho p38 MAP kinase monoclonal antibody, measurement of activity by phosphorylation of ATF 2 and detection of phosphorylated ATF 2 by immunoblotting using anti phospho ATF 2 and densitometric analysis using a FOTOAnalyst imaging system (Fotodyne, Hartland, WI) and TotalLab imaging software (TotalLab, Newcastle-upon-Tyne, England, UK).

### Immunoblot Analysis of ERK 1/2 and p38 MAP Kinases

Urothelial cells were suspended in lysis buffer containing (mmol/l) HEPES 20 (pH 7.6), sucrose 250, dithiothreitol 2, EDTA 2, EGTA 2, β-glycerophosphate 10, sodium orthovanadate 1, phenylmethylsulfonyl fluoride 2, leupeptin 20 µg/ml, aprotinin 10 µg/ml and pepstatin A 5 µg/ml. Cells were sonicated on ice and centrifuged at 20,000×g at 4°C for 20 min to remove cellular debris and nuclei. Cytosolic protein was separated by SDS/PAGE and electrophoretically transferred to PVDF membranes (Bio-Rad, Richmond, CA). The blocked PVDF membrane was incubated with primary antibodies to phosphorylated MAP kinases and horseradish peroxidase-conjugated secondary antibodies. Regions of antibody binding were detected using enhanced chemiluminescence (Amersham, Arlington Heights, IL) after exposure to film (Hyperfilm, Amersham). Equal loading was verified by immunoblot analysis for total MAP kinase protein in each lane.

### Phospholipase A_2_ Activity

Urothelial cell cultures were washed with ice-cold PBS and suspended in 1 ml PLA_2_ assay buffer containing (mmol/l): Sucrose 250, KCl 10, imidazole 10, EDTA 5, dithiothreitol (DTT) 2 with 10% glycerol, pH = 7.8. The suspension was sonicated and the sonicate centrifuged to remove cellular debris and nuclei. Phospholipase A_2_ activity in the supernatant (cytosolic and membrane protein) was assessed by incubating cellular protein (50 µg) with 100 µM (16∶0, [^3^H]18∶1) plasmenylcholine substrate in buffer containing (mmol/l): Tris 10, EGTA 4, 10% glycerol, pH = 7.0 at 37°C for 5 minutes in a total volume of 200 µl. Radiolabeled plasmenylcholine was synthesized as described previously [26]. Reactions were terminated by the addition of 100 µl butanol and released radiolabeled fatty acid was isolated by application of 25 µl of the butanol phase to channeled Silica Gel G plates, development in petroleum ether/diethyl ether/acetic acid (70/30/1, v/v) and subsequent quantification by liquid scintillation spectrometry.

### Measurement of Wound Healing of Confluent Urothelial Cell Cultures

Wound healing and repair in urothelial cells was determined using the electric cell-substrate impedance sensing (ECIS) system (Applied Biophysics, Troy, NY) as described previously [27]. Urothelial cells from normal and IC/PBS bladders were grown on ECIS electrode arrays (8W1E). The impedance fluctuations of cell attachment and spread were continuously monitored. An alternating current of 1 *µ*A at 4 kHz was applied between a small sensing electrode (250-*µ*m diameter) and a relatively large counter electrode. Impedance measurements were analyzed at 5 minute intervals and verified that confluence was achieved. At confluence, 10% fetal bovine serum (FBS) and additional 1.0 mM calcium was added to the culture medium. All experiments were conducted 3 days after calcium and FBS addition. Wounding of urothelial cells was achieved using a 6V signal at 45 kHz for a duration of 30 sec. Application of this field results in a rapid drop in the impedance of cell layers due to the cell death of the cells on the electrode. Impedance increases as cells migrate from the perimeter of the electrode inward to replace the killed cells.

### Statistical Analysis

Statistical significance between multiple values was determined using one-way analysis of variance with Dunnett’s test *post-hoc* analysis. All results are expressed as means±S.E.M. Statistical significance was considered to be p<0.05.

## Results

**Figure 1:**
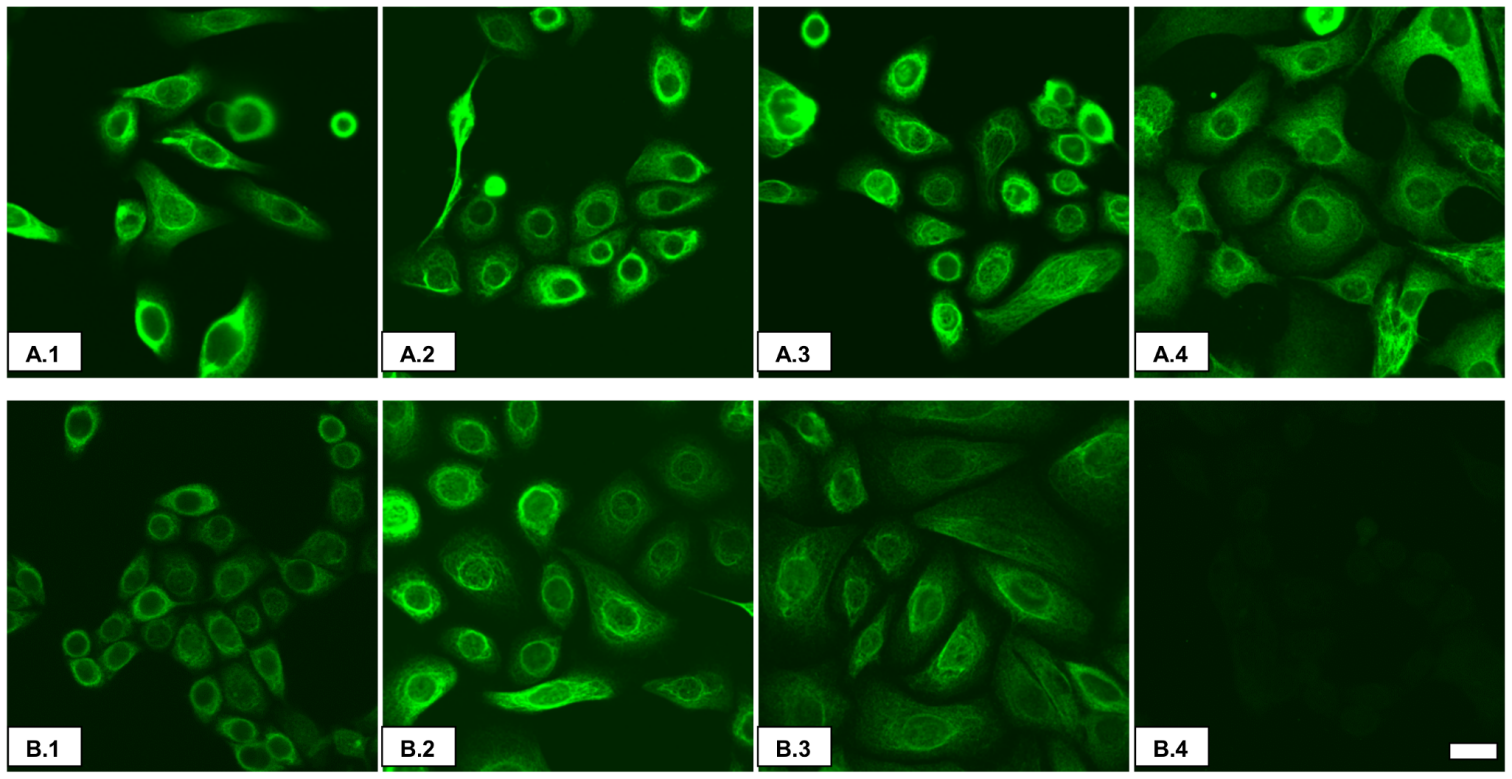
Cytokeratin expression of immortalized urothelial cells. **Row A:** A.1.HUC line. A.2–A.4. Representative immortalized cell lines from normal patients. **Row B:** B.1–B.3. Representative IC/PBS patient-derived immortalized cell lines. B.4. Representative background. Magnification 60×. Magnification Scale Bar = 20 µm.

Human urothelial cells (HUC, A.1 in Figure 1) and immortalized urothelial cells from normal (A.2–A.4, Figure 1) and IC/PBS (B.1–B.3, Figure 1) bladders were stained for epithelial cytokeratin AE1/AE3. All samples used in subsequent experiments stained positively when compared to background staining (B.4, Figure 1).

**Figure 2:**
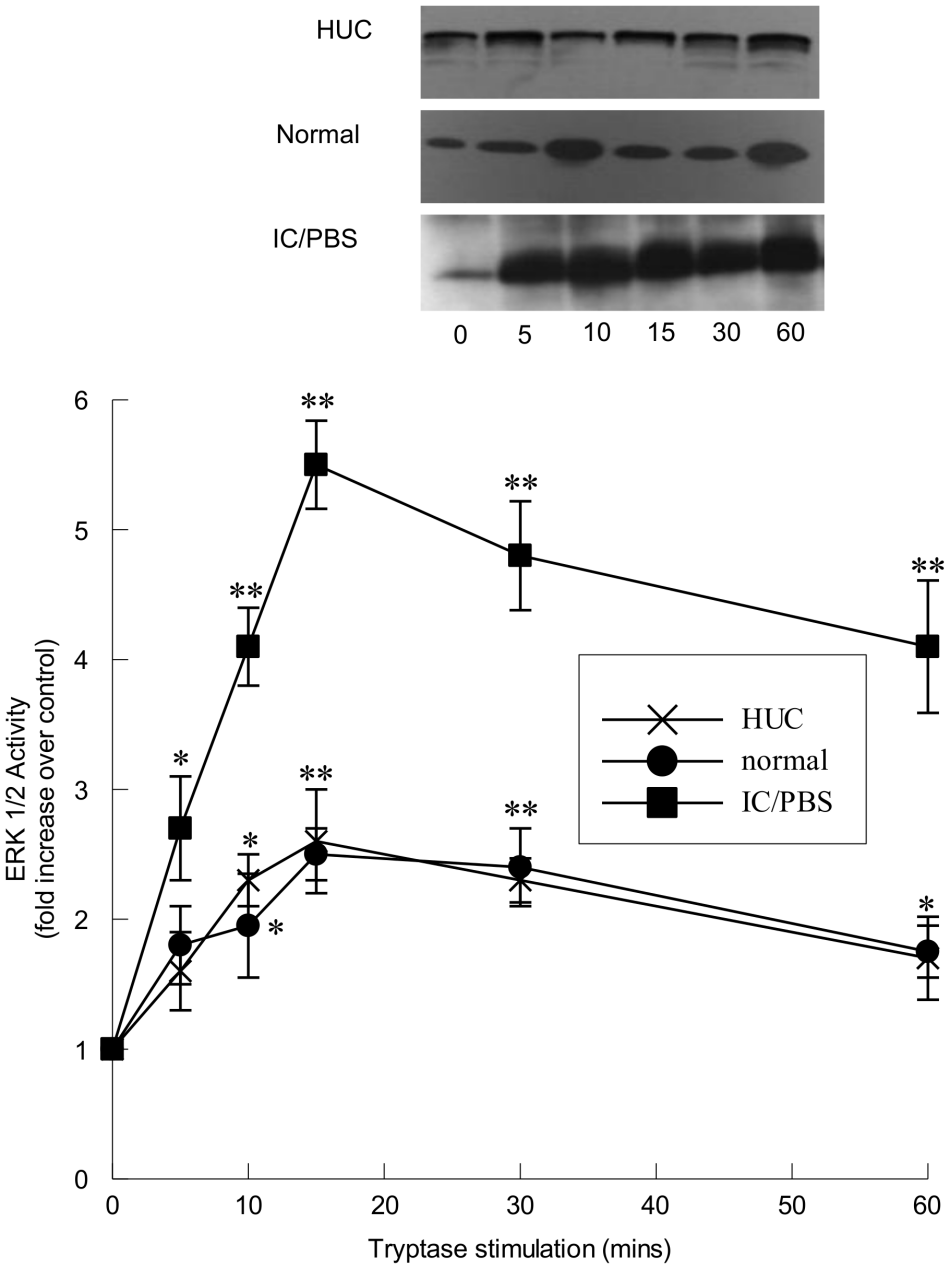
ERK 1/2 activation within immortalized urothelial cells following tryptase stimulation. Upper panel: Representative immunoblots of ERK 1/2 activity measured as Elk-1 phosphorylation in tryptase-stimulated (20 ng/ml) human urothelial cells (HUC) and immortalized urothelial cells from normal and IC/PBS patients. Lower panel: ERK 1/2 activity, measured by densitometry of immunoblots represented in upper panel, was significantly increased in tryptase-stimulated HUC (X) and immortalized cells from normal (filled circles) and IC/PBS bladders (filled squares). ERK 1/2 activity is expressed as a fold increase over unstimulated values and was determined from 25 µg cytosolic protein. *p<0.05, **p<0.01 when compared to unstimulated activity. Data shown are mean±SEM for results from 4 different experiments using cell isolations from 3 (HUC) donors or 4 separate patients (immortalized cells from normal and IC/PBS bladder).

Urothelial cells were stimulated with tryptase (20 ng/ml) for up to 60 mins and MAP kinase activity was measured. Tryptase stimulation of immortalized urothelial cells from normal bladders (Figure 2) resulted in a 2- to 3-fold increase in extracellular signal-regulated kinase 1/2 (ERK 1/2) activity that occurred within 15 mins of stimulation and remained elevated over 60 min. However, tryptase stimulation of immortalized urothelial cells from IC/PBS patients (Figure 2) resulted in a much greater increase in ERK 1/2 activation when compared with that of normal urothelial cells at all time points measured. Activation of ERK 1/2 was similar between primary human urothelial cells (HUC) and immortalized urothelial cells indicating the immortalization procedure did not affect responses in normal cells (Figure 2).

**Figure 3:**
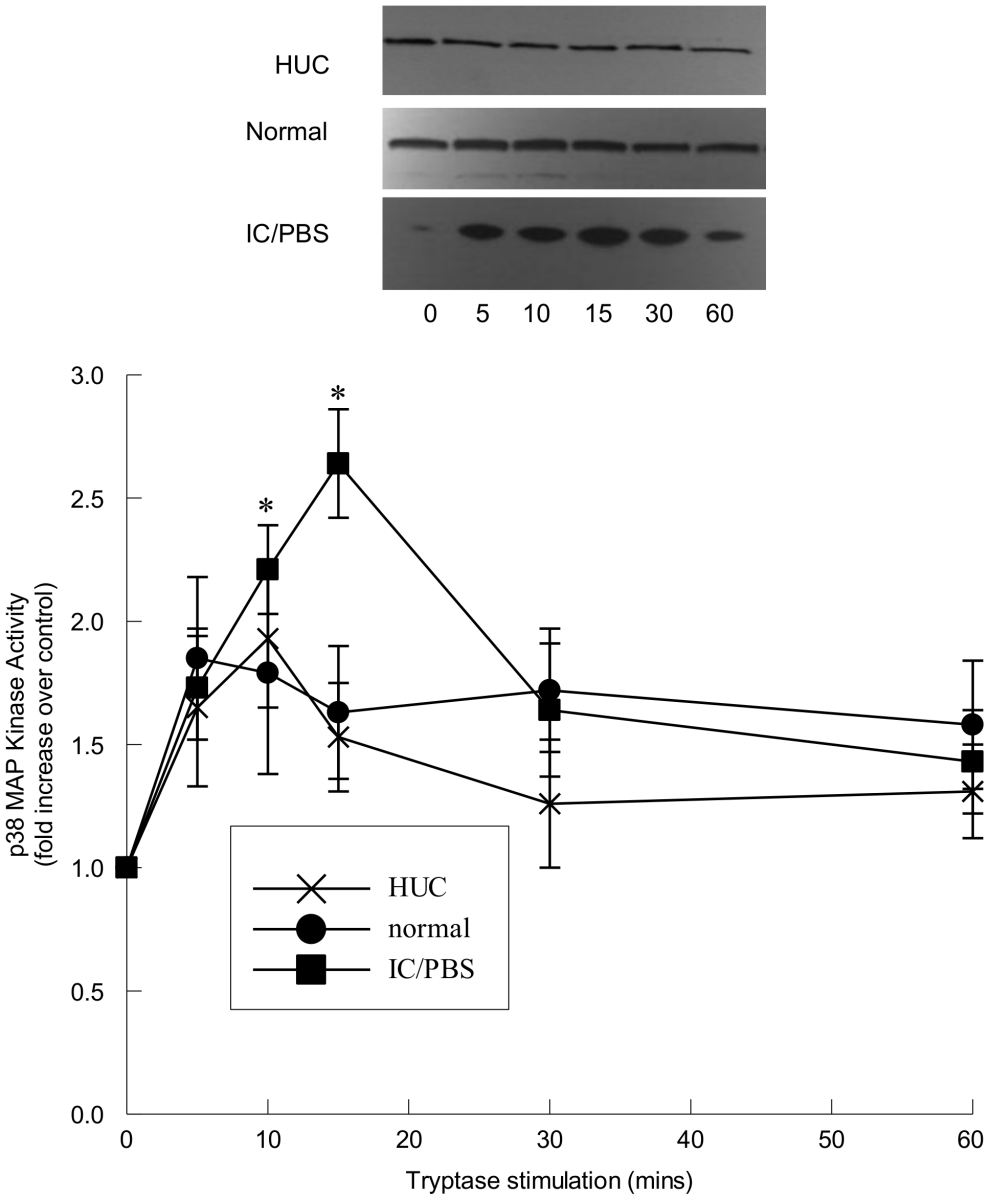
p38 MAP Kinase activation within immortalized urothelial cells following tryptase stimulation. Upper panel: Representative immunoblots of p38 MAP kinase measured as ATF-2 phosphorylation in tryptase-stimulated (20 ng/ml) human urothelial cells (HUC) and immortalized cells from normal and IC/PBS patients. Lower panel: p38 MAP kinase activity was significantly increased in tryptase-stimulated HUC (X) and immortalized cells from normal (filled circles) and IC/PBS bladders (filled squares). p38 MAP kinase activity is expressed as a fold increase over unstimulated values and was determined from 25 µg cytosolic protein. Data shown are mean±SEM for results from 4 different experiments using cell isolations from 3 (HUC) donors or 4 separate patients (immortalized cells from normal and IC/PBS bladder).

Tryptase stimulation (20 ng/ml) of primary (Figure 3) and immortalized (Figure 3) urothelial cells resulted in a small, but significant, increase in p38 MAP kinase activity. Similarly, activation of urothelial cells from IC/PBS patients with tryptase demonstrated an increase in p38 MAP kinase activity (Figure 3). Although there was a significant increase in p38 MAP kinase activity following tryptase stimulation, the increased p38 activity was brief and not as sustained as the ERK 1/2 activity.

**Figure 4:**
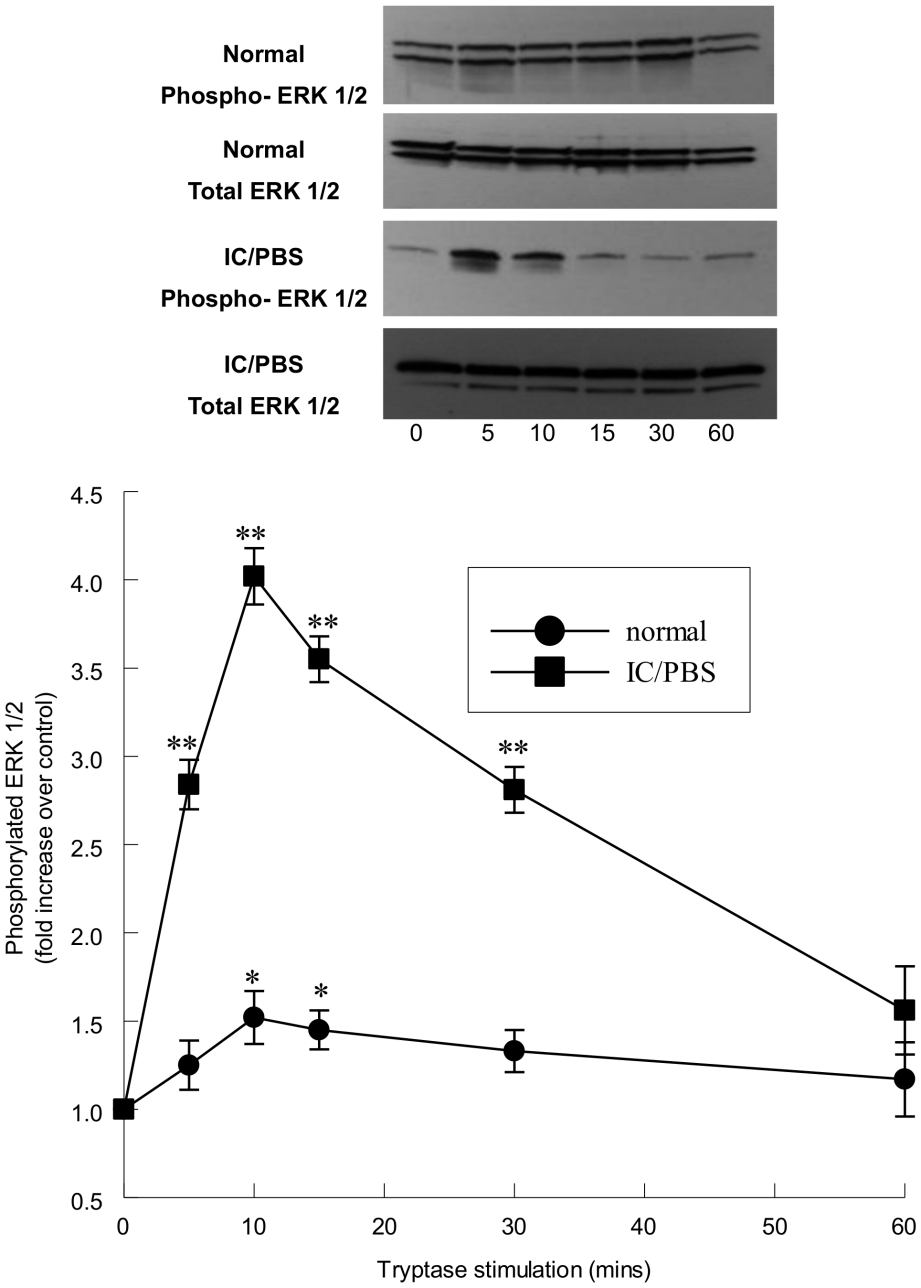
Phosphorylation of ERK1/2 in urothelial cells following tryptase stimulation. Upper panel: Representative immunoblots of phosphorylated and total ERK 1/2 in normal and IC/PBS urothelial cells stimulated with tryptase (20 ng/ml). ERK 1/2 activity was significantly increased in tryptase-stimulated immortalized cells from normal (filled circles) and IC/PBS bladders (filled squares). ERK 1/2 activity is expressed as a fold increase over unstimulated values. *p<0.05, **p<0.01 when compared to unstimulated activity. Data shown are mean±SEM for results from 4 different experiments using cell isolations from 4 separate patients or donors.

To determine whether tryptase-stimulated ERK 1/2 activation was a result of phosphorylation of the enzyme, we performed immunoblot analysis for phosphorylated ERK 1/2 and normalized its expression to total ERK 1/2 in urothelial cells. Tryptase stimulation of normal urothelial cells resulted in a 1.5-fold increase in phosphorylated ERK 1/2 (Figure 4). A 4-fold increase in phosphorylated ERK 1/2 was observed in tryptase-stimulated urothelial cells from IC/PBS patients (Figure 4).

**Figure 5:**
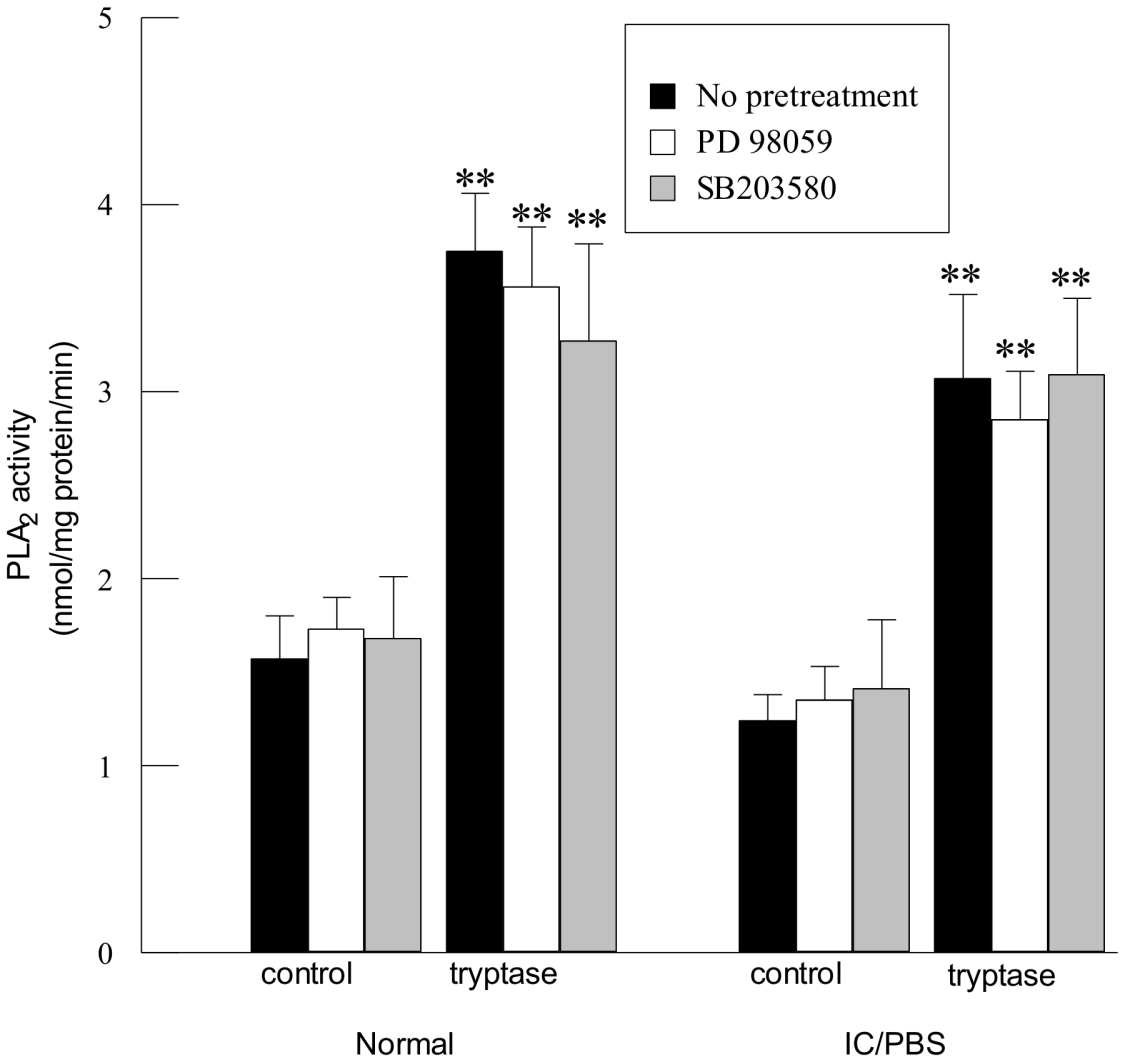
Inhibition of MAP kinases does not affect urothelial cell iPLA_2_ activity. Pretreatment of immortalized urothelial cells from normal or IC/PBS bladders with PD98059 (5 µM, 10 min, open bars) or SB203580 (1 µM, 10 min, grey bars) had no effect on tryptase-stimulated (20 ng/ml, 5 mins) calcium-independent phospholipase A_2_ (iPLA_2_) activity (black bars). Data shown are mean±SEM for results from 3 different experiments using cell isolations from 4 separate patients or donors. **p<0.01 when compared to corresponding unstimulated values.

We have previously determined that tryptase stimulation of urothelial cells results in activation of calcium-independent phospholipase A_2_ (iPLA_2_) [24]. To determine whether activation of iPLA_2_ is mediated by MAP kinase, we pretreated urothelial cells with PD 98059 to inhibit ERK 1/2 or SB 203580 to inhibit p38 MAP kinase. Tryptase stimulation (20 ng/ml, 5 mins) of normal or IC/PBS urothelial cells resulted in activation of iPLA_2_ (Figure 5). Pretreatment with PD 98059 or SB 203580 (Figure 5) had no significant effect on tryptase-stimulated iPLA_2_ activity in either control or tryptase-stimulated urothelial cells, indicating that MAP kinase does not mediate iPLA_2_ activation.

**Figure 6:**
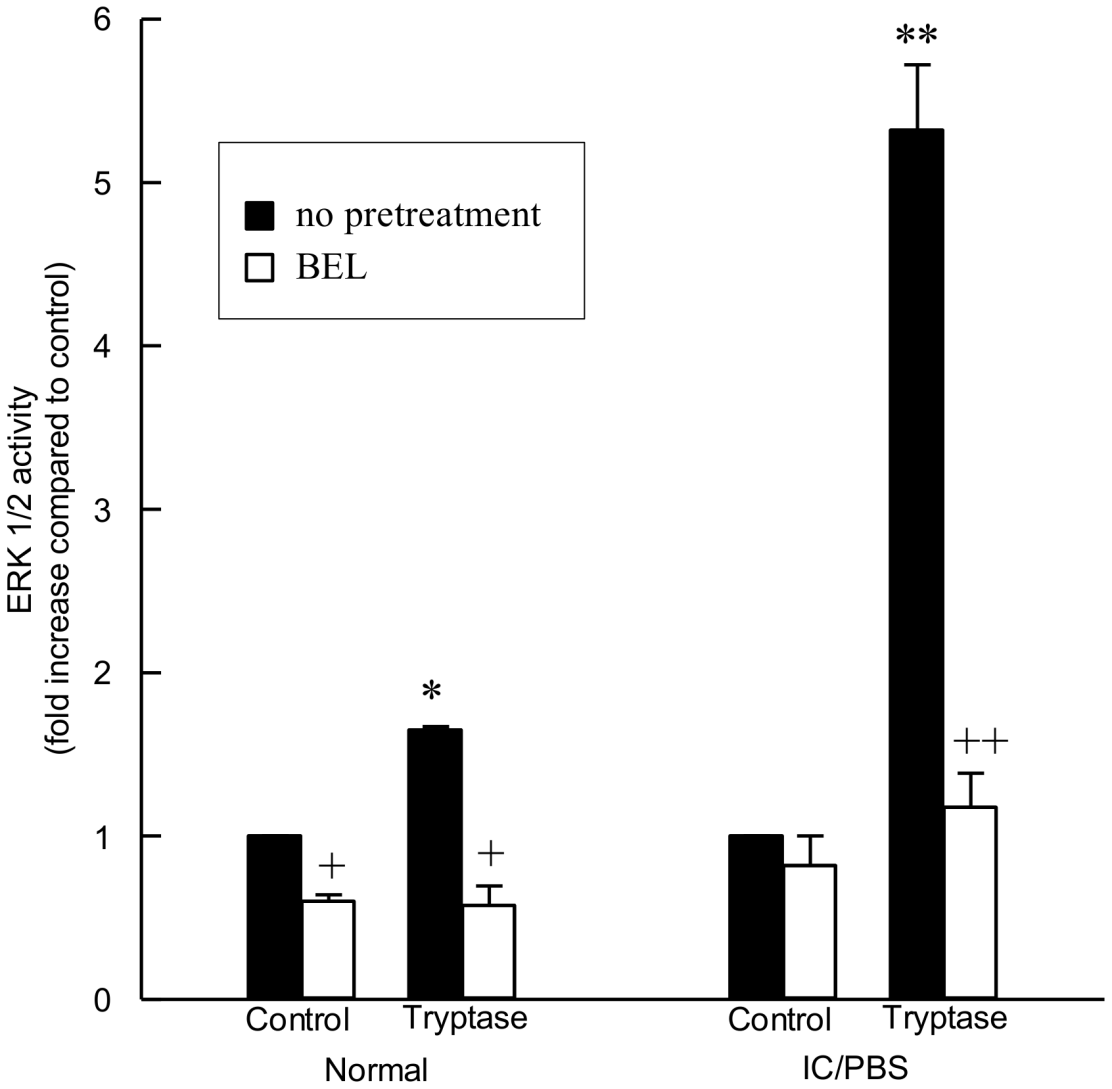
Inhibition of iPLA_2_ significantly inhibits tryptase stimulated ERK 1/2 activation. Pretreatment of immortalized urothelial cells from normal or IC/PBS bladders with bromoenol lactone (BEL, 5 µM, 10 mins, open bars) significantly inhibited tryptase-stimulated (20 ng/ml, 10 mins) ERK 1/2 activation (black bars). Data shown are mean+SEM for results from 3 different experiments using cell isolations from 4 separate patients or donors. *p<0.05, **p<0.01 when compared to corresponding unstimulated values. ^+^p<0.05, ^++^p<0.01 when comparing corresponding values in the presence or absence of BEL.

**Figure 7:**
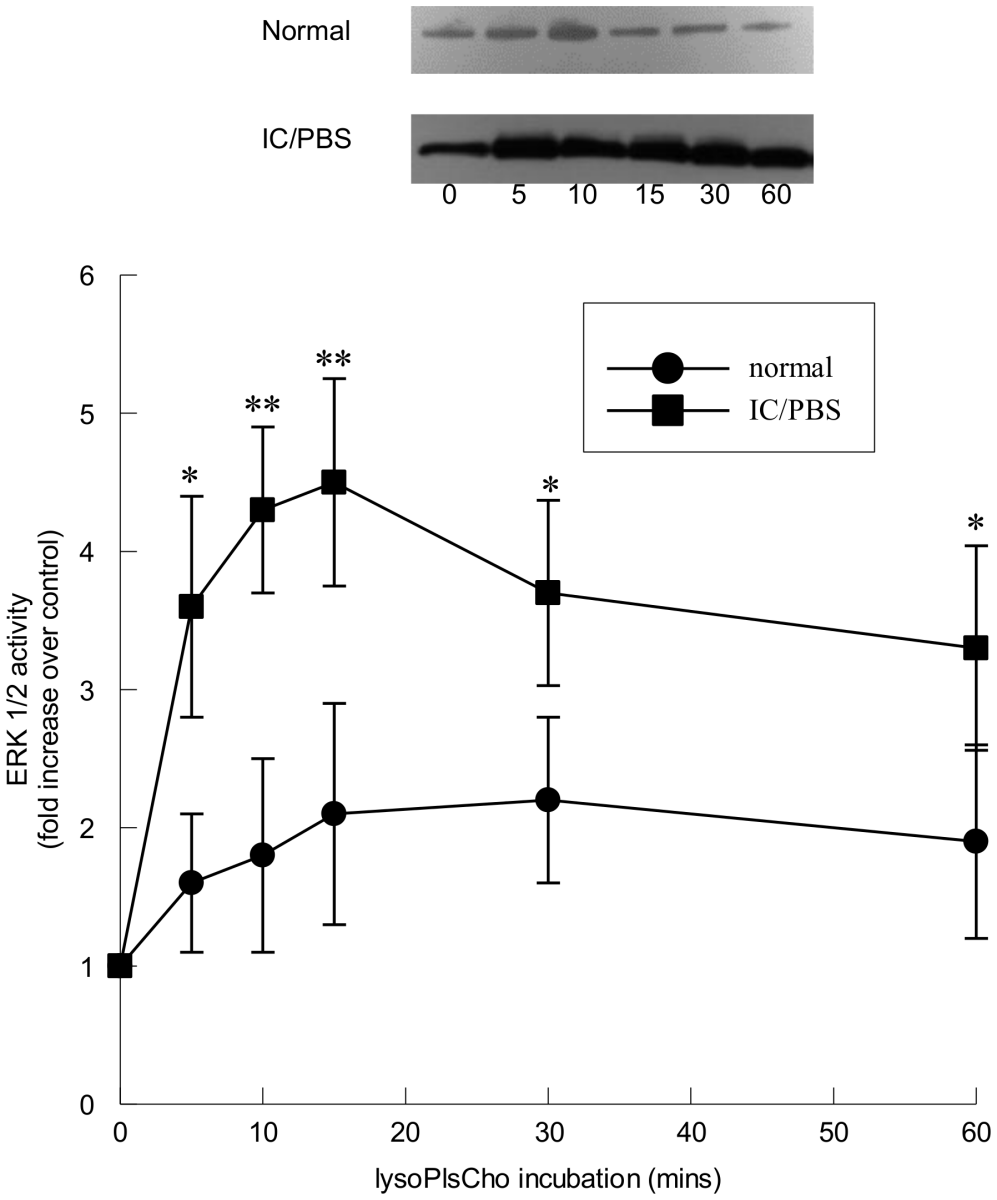
ERK 1/2 activation of immortalized urothelial cells following stimulation with lysoplasmenylcholine. Upper panel: Representative immunoblots of ERK 1/2 activity measured as Elk-1 phosphorylation in lysoplasmenycholine-stimulated (lysoPlsCho, 5 µM) immortalized urothelial cells from normal and IC/PBS patients. Lower panel: ERK 1/2 activity was activated by lysoPlsCho in immortalized urothelial cells from IC/PBS bladders (filled squares) when compared to normal bladders (filled circles). ERK 1/2 activity is expressed as a fold increase over unstimulated values and was determined from 25 µg cytosolic protein. *p<0.05, **p<0.01 when compared to unstimulated activity. Data shown are mean±SEM for results from 6 different cell cultures.

To determine if ERK 1/2 activation was downstream of iPLA_2_, we pretreated urothelial cells with bromoenol lactone (BEL, 5 µM, 10 mins) prior to tryptase stimulation (Figure 6). Pretreatment with the iPLA_2_-selective inhibitor BEL (Figure 6) completely inhibited tryptase-stimulated ERK 1/2 (Figure 6, black bars) in both normal and IC/PBS urothelial cells, suggesting that ERK 1/2 activation is downstream of iPLA_2_ in tryptase-stimulated urothelial cells. Phospholipase A_2_ hydrolyzes the *sn*-2 fatty acid of membrane phospholipids, resulting in the release of a lysophospholipid and a free fatty acid, most importantly arachidonic acid. We have previously demonstrated that urothelial cell iPLA_2_ activity is selective for plasmalogen phospholipids [24]. To determine whether ERK 1/2 activation is mediated by one or both of the metabolites of iPLA_2_-catalyzed membrane phospholipid hydrolysis, we incubated urothelial cells with lysoplasmenylcholine (lysoPlsCho, 5 µM) for up to 60 min. A significant increase in ERK 1/2 activity was observed in IC/PBS urothelial cells incubated with lysoPlsCho after 5 min (Figure 7). A small, but not significant, increase in ERK 1/2 activity was observed in normal urothelial cells incubated with lysoPlsCho (Figure 7). Urothelial cells were incubated with 5 µM arachidonic acid for up to 60 min, but no increase in ERK 1/2 activity was observed (data not shown).

**Figure 8:**
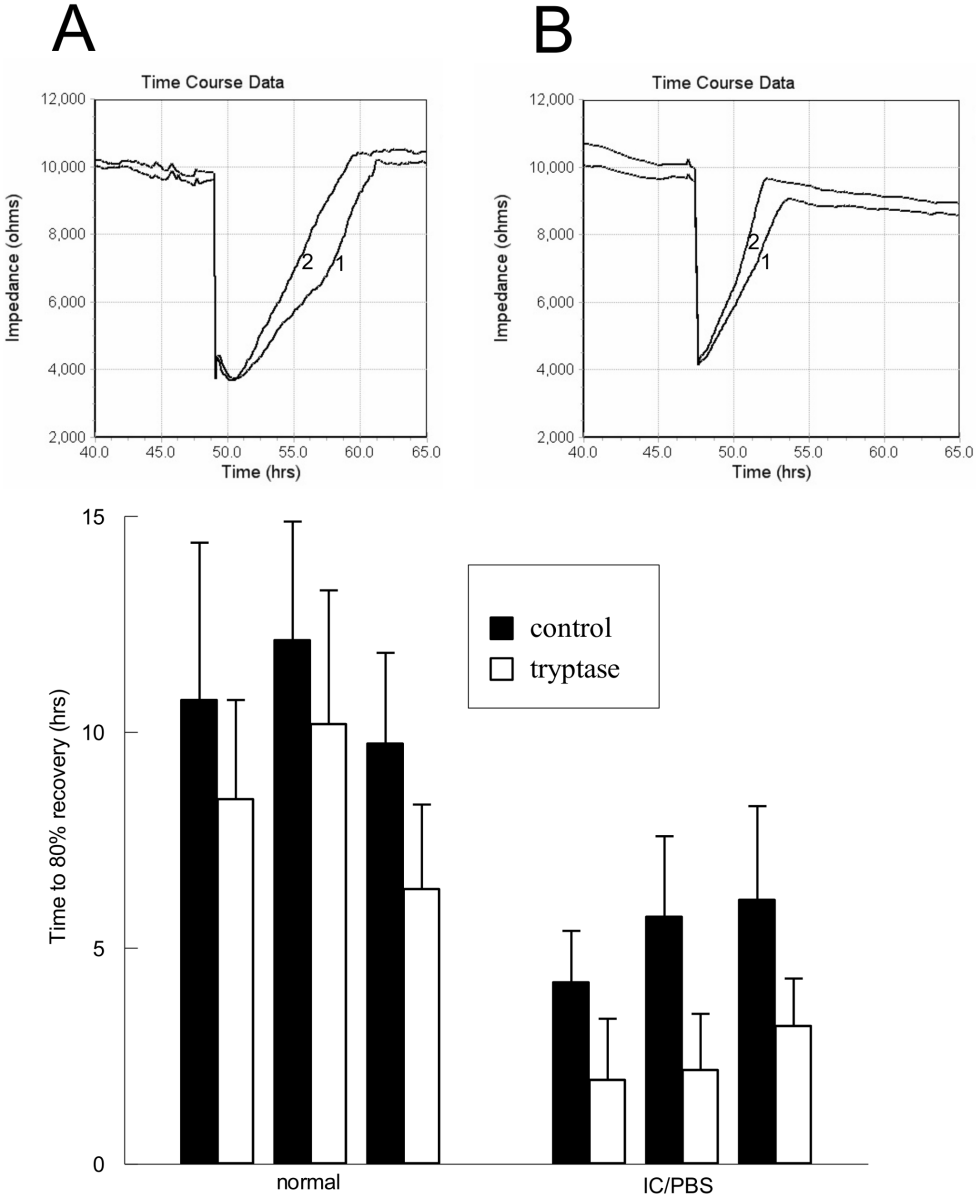
Incubation of urothelial cells with tryptase results in increased rate of urothelial cell wound healing. Upper Panel: Wound healing of immortalized urothelial cells from a normal bladder (8A, line 1) and from an IC/PBS bladder (8B, line 1) is accelerated in the presence of tryptase (20 ng/ml, Panels A and B, line 2). Lower Panel: Time to recover impedance to 80% of pre-wounding values is consistently reduced in the presence of tryptase (20 ng/ml) in normal and IC/PBS urothelial cells. Data shown are mean±SEM for results from 4 different cell cultures from 4 cell isolations from normal or IC/PBS patients.

Enhanced ERK 1/2 activation could contribute to enhanced cell proliferation or differentiation in urothelial cells. To determine whether tryptase stimulation resulted in increased wound healing rates, we cultured urothelial cells isolated from normal or IC/PBS bladders on ECIS electrodes and measured impedance across the cells in real time. Wounding of the cells resulted in an immediate decrease in measured resistance that returned towards normal values over time (Figures 8A and 8B). Recovery of pre-electroporation impedance was consistently more rapid but not statistically significant in urothelial cells from IC/PBS patients (Figure 8B, line 1) than in those isolated from normal patients (Figure 8A, line 1). Incubation with tryptase (20 ng/ml, line 2 in Figures 8A and 8B) reduced the time for impedance recovery in both normal and IC/PBS urothelial cells (Figure 8, lower panel, open bars) when compared to impedance recovery in the absence of tryptase (Figure 8, lower panel, closed bars).

**Figure 9:**
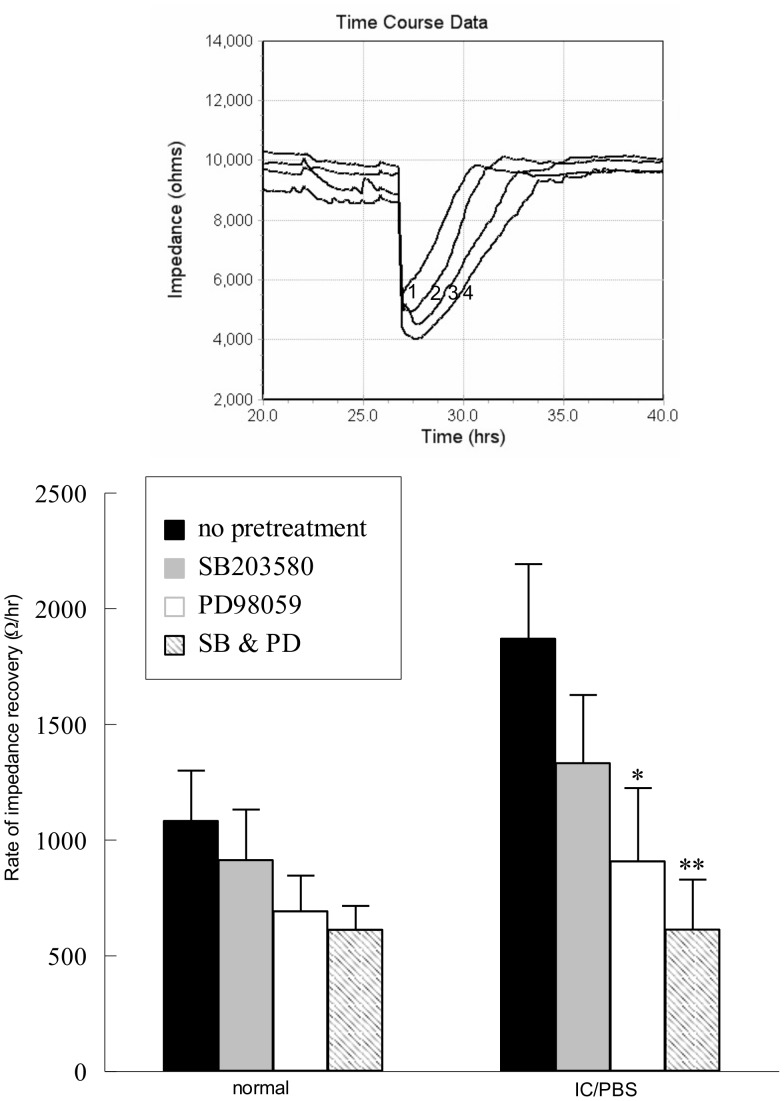
Rate of wound healing is significantly reduced in the presence of ERK 1/2 inhibition. Upper Panel: Rate of wounding recovery in immortalized urothelial cells from an IC/PBS patient bladder (trace 1) was decreased in the presence of SB203580 (1 µM, trace 2), PD95089 (2 µM, trace 3) and a combination of PD98059 and SB203580 (trace 4). Lower panel: Rate of wound healing is significantly decreased when immortalized urothelial cells are exposed to PD98059 (2 µM) with or without SB203580 (1 µM). Data shown are mean+SEM for results from 4 different cell cultures from 4 cell isolations from normal or IC/PBS patients. *p<0.05, **p<0.01 when compared to wound healing in the absence of inhibitor.

To determine whether wound healing was mediated via MAP kinase activation, tryptase-stimulated urothelial cells were pretreated with PD98059 (2 µM, Figure 9, upper panel, line 3), SB203580 (1 µM, Figure 9, upper panel, line 2) or a combination of both (Figure 9, upper panel, line 4). The rate of impedance recovery to 80% of baseline was not significantly increased by MAP kinase inhibition in urothelial cells isolated from normal bladders (Figure 9, lower panel). However, inhibition of ERK 1/2 by pretreatment with PD98059 significantly delayed wound healing rates in urothelial cells isolated from IC/PBS bladders, which was further inhibited by the addition of SB203580 to inhibit p38 MAP kinase (Figure 9).

Taken together, these data demonstrate a significant potentiation of ERK 1/2 activation in tryptase-stimulated urothelial cells isolated from the bladders of IC/PBS patients when compared to those from normal bladders. Activation of ERK 1/2 is downstream of, and dependent upon, tryptase-stimulated iPLA_2_ activity. Enhanced ERK 1/2 activity may contribute to enhanced wound healing rate in IC/PBS patients.

## Discussion

In the present study, we have demonstrated that urothelial cell ERK 1/2 is activated in response to tryptase stimulation and is downstream of iPLA_2_ activation. Additionally, ERK 1/2 activation is significantly greater in immortalized urothelial cells isolated from the inflamed areas of IC/PBS bladders when compared to cells isolated from normal bladders. In a subset of patients with IC/PBS, increased mast cell numbers and activation in the bladder wall are implicated in the pathogenesis of the disease [3]. Several inflammatory mediators are released upon mast cell activation and increased urinary concentrations of interleukin-6 (IL-6), histamine, leukotrienes and tryptase have been observed in IC/PBS patients [3]. Tryptase activates the protease-activated receptor-2 (PAR-2) on the surface of urothelial cells, leading to activation of iPLA_2_ and the production of membrane phospholipid-derived inflammatory mediators [18], [24].

We have determined that tryptase stimulates urothelial cell ERK 1/2 activity and that the response is greater in urothelial cells isolated from IC/PBS bladders. Activation of the ERK signaling pathway has been implicated in several cellular functions, including differentiation, proliferation and inflammatory responses. Activation of MAP kinases is a frequent event in tumor progression and has been implicated in the log phase growth of bladder cancer [28]. However, Swiatkowski et al [29] demonstrated that proliferation of urothelial cancer cells was much less dependent on MAP kinase activation than normal urothelial cell proliferation. Thus, to determine that the immortalization procedure did not alter MAP kinase responses to tryptase, we compared MAP kinase activation between primary human bladder urothelial cells and immortalized urothelial cells isolated from normal bladders. We determined that activation of MAP kinases in response to tryptase stimulation were similar between primary and immortalized normal urothelial cells, suggesting that the immortalization procedure did not affect MAP kinase activation.

ERK 1/2 activation could contribute to enhanced cell proliferation or differentiation in the IC/PBS bladder, however, IC/PBS is associated with thinning and ulceration of the bladder wall. This apparent discrepancy could be due to the presence of antiproliferative factor (APF) that has been reported in the urine of IC/PBS patients [30]. APF has been shown to decrease the proliferation of normal bladder urothelial cells by antagonizing the normal ERK activation cascade by heparin-binding epidermal growth factor-like growth factor and instead activating p38 MAP kinase [31]. These data are consistent with activation of ERK 1/2 pathways being associated with cell proliferation whereas activation of p38 MAP kinase is generally associated with inhibition of cell growth [28]. In our studies, we did not observe significant increases in p38 MAP kinase activity in tryptase-stimulated immortalized urothelial cells from normal patients. However, significant increases in p38 MAP kinase activity were observed in IC/PBS immortalized urothelial cells. A recent study by Shie et al [10] has determined that there is enhanced phosphorylated p38 MAP kinase in the bladder of IC/PBS patients when compared to controls and the authors suggest that this is indicative of increased inflammation in the IC/PBS bladder. Our results obtained from *in vitro* studies agree with their findings in clinical samples.

Wang et al [32] have demonstrated an increase in COX-2 expression in response to PAR-2 activation in human urothelial cells that is mediated primarily through the ERK 1/2 MAP kinase pathway. We have previously demonstrated increased PGE_2_ release was abrogated in IC/PBS urothelial cells relative to normal cells [18]. Thus, the increased ERK 1/2 activation in IC/PBS urothelial cells when compared to normal bladder urothelial cells did not result in enhanced PGE_2_ production as might be expected otherwise. In our previous study [18], we determined that the failure to produce PGE_2_ in response to tryptase was due to a decrease in mRNA for COX-2 and PGE synthase, together with increased expression of 15-hydroxyprostaglandin dehydrogenase, suggesting that both impaired synthesis and increased catabolism contributes to the lack of PGE_2_ production in urothelial cells from IC/PBS bladders.

There are many changes in the bladders of IC/PBS patients that may affect the urothelium. A recently published study [33] used urinary proteomics for normal and IC/PBS patients and identified several proteins and pathways that may contribute to IC/PBS pathology. Data from our laboratory and others suggests that there are biochemical changes that could enhance or impede cell proliferation and differentiation. In IC/PBS, the secretion of APF and the loss of PGE_2_ release could both contribute to impairment of urothelial repair and barrier function, whereas the data presented here suggest that ERK 1/2 activation may enhance these processes. It is possible that enhanced ERK 1/2 activation in IC/PBS urothelial cells is an adaptive response to other biological changes in the diseased urothelium. Our *in vitro* studies indicated enhanced cell proliferation in IC/PBS urothelial cells compared to normal. These data agree with urinary proteomics studies that have identified upregulation of proteins involved in responses to wounding in IC/PBS patients [33]. *In vivo*, the secretion of APF, or other adaptive responses, may negate any beneficial effect of enhanced ERK 1/2. Taken together, data obtained in this study and others demonstrate the importance of acknowledging that several adaptive processes may occur in IC/PBS that may work in concert or against each other and represent a delicate balance between protection and damage to the bladder wall. These changes not only survive in culture, they survive immortalization.

In summary, the activation of G protein-coupled receptors, such as PAR-2 results in increased phospholipase activity, synthesis of membrane phospholipid-derived second messengers and activation of phosphorylation events that can lead to mitogenic signals and cell proliferation. Activation of the urothelial cell ERK 1/2 pathway by tryptase released from activated mast cells may represent a beneficial response in IC/PBS and be associated with the facilitation of wound healing or cell proliferation in areas of inflammation.
